# Outcomes of patients treated with SVILE *vs.* P-GemOx for extranodal natural killer/T-cell lymphoma, nasal type: a prospective, randomized controlled study

**DOI:** 10.20892/j.issn.2095-3941.2020.0160

**Published:** 2020-08-15

**Authors:** Liqiang Wei, Lei Yang, Jin Ye, Jia Cong, Xin Li, Na Yao, Jing Yang, Xueying Cui, Jing Ding, Yiping Wu, Jingwen Wang

**Affiliations:** ^1^Department of Hematology, Beijing Tongren Hospital, Capital Medical University, Beijing 100730, China

**Keywords:** Extranodal natural killer/T-cell lymphoma, nasal type, chemotherapy, overall response, radiotherapy

## Abstract

**Objective:** To compare the efficacy and safety of the novel SVILE regimen with the P-GemOx regimen in patients with newly diagnosed extranodal natural killer/T-cell lymphoma, nasal type (ND-ENKTL).

**Methods**: From April 2015 to July 2018, 103 patients with ND-ENKTL were randomly assigned to SVILE (experimental group) or P-GemOx (control group) chemotherapy followed by radiotherapy and consolidation chemotherapy. The primary endpoint was the overall response rate after 3 cycles of chemotherapy, and secondary study endpoints were complete response (CR), progression-free survival (PFS), and overall survival (OS). Safety was also evaluated.

**Results:** There were no significant differences in baseline characteristics in the experimental *vs.* control groups. In experimental and control groups, respectively, the overall response rates were 91.7% *vs.* 97.0% for stage I/II and 75.0% *vs.* 72.2% for stage III/IV. The CR rates were 83.4% *vs.* 97.0% for stage I/II and 68.8% *vs.* 61.1% for stage III/IV. None of those differences were significant. There was no significant difference in PFS and OS between groups and between patients in stage I/II and stage III/IV. The 3-year PFS and OS in stage I/II were 88.3% *vs.* 93.3% and 88.8% *vs.* 97.0%, respectively. The 3-year PFS and OS in stage III/IV were 46.2% *vs.* 65.7% and 68.8% *vs.* 72.2%, respectively. The common adverse events were hematological toxicity, hepatotoxicity, and coagulation abnormalities, which were found to be reversible with supportive therapy.

**Conclusions:** The novel SVILE regimen has comparable effects to those of P-GemOx in patients with ND-ENKTL and is well tolerated. SVILE is a therapeutic option for ND-ENKTL.

## Introduction

According to the World Health Organization classification of tumors in hematopoietic and lymphoid tissues, extranodal natural killer/T-cell lymphoma (ENKTL) is a tumor of mature T- and NK-cells that is closely associated with Epstein-Barr virus infection^1^. In Western countries, ENKTL accounts for a relatively small number of all non-Hodgkin lymphoma cases^2^; however, ENKTL is relatively common in China, accounting for 12.0%–17.1% of non-Hodgkin lymphoma cases^3,4^. Currently, although considerable progress has been made in the treatment of ENKTL, owing to the relatively poor results, anthracycline-containing regimens, such as cyclophosphamide, doxorubicin, vincristine, and prednisone (CHOP) and CHOP plus etoposide, are no longer considered ideal for the treatment of ENKTL^5^, because the treatment options have been improved. Asparaginase-based combined chemotherapy regimens, such as gemcitabine, dexamethasone, and cisplatin (GDP); pegaspargase, gemcitabine, and oxaliplatin (P-GemOx); GemOx; and modified dexamethasone, methotrexate, ifosfamide, asparaginase, and etoposide (mSMILE) result in better outcomes than those of prior treatment protocols and have now become mainstream chemotherapy regimens for the treatment of ENKTL^6–11^. Furthermore, monoclonal antibodies to programmed cell death protein 1 and programmed cell death ligand 1 have emerged as promising treatment options for refractory or relapsed ENKTL^12–14^. Studies have also shown promising results of treatments with monoclonal antibodies against cluster of differentiation (CD)52 and the protein deacetylase inhibitor romidepsin^15,16^. Furthermore, many molecular targets for ENKTL are being introduced^17^. Although newer regimens have demonstrated positive outcomes in the treatment of ENKTL, they still do not always meet patients’ needs. Many guidelines, including those of the National Comprehensive Cancer Network, emphasize the necessity for new clinical trials examining the treatment of newly diagnosed ENKTL (ND-ENKTL). Therefore, we designed SVILE (ifosfamide, dexamethasone, pegaspargase, vindesine, and etoposide), a novel chemotherapy regimen for ENKTL treatment. In this prospective controlled study, SVILE was used in the experimental group, the commonly used P-GemOx treatment was used in the control group, and induction chemotherapy + involved field radiotherapy (IFRT) + consolidation chemotherapy was the treatment protocol for participants who responded effectively to induction chemotherapy. Short-term and long-term efficacy and adverse events (AEs) in the groups of patients receiving ND-ENKTL were compared. We hypothesized that the SVILE regimen would have similar or better efficacy than the P-GemOx regimen in the treatment of ENKTL, and would potentially provide new treatment options for patients with ENKTL.

## Materials and methods

### Treatment protocol and patients

**Figure 1:**
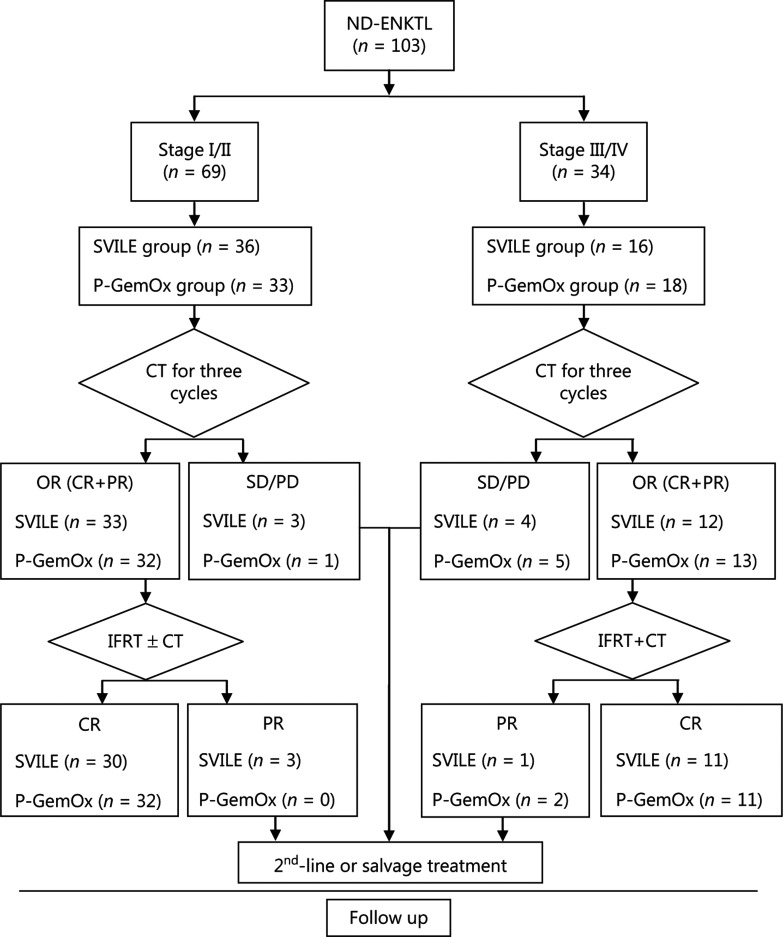
Study design and treatment protocol. ND-ENKTL, newly diagnosed extranodal natural killer/T-cell lymphoma, nasal type; SVILE, ifosfamide, dexamethasone, pegaspargase, vindesine, and etoposide; P-GemOx, pegaspargase, gemcitabine, and oxaliplatin; CT, computed tomography; ORR, overall response rate; CR, complete response; PR, partial response; SD, stable disease; PD, progressive disease; IFRT, involved field radiotherapy.

This was a prospective, stratified randomized controlled study. Enrolled patients were stratified into stage I/II or stage III/IV, and randomly assigned to the experimental group (SVILE treatment group) or the control group (P-GemOx treatment group). The overall response rate (ORR) was evaluated after 3 cycles of induction chemotherapy, and patients who responded effectively received chemotherapy + IFRT + consolidation chemotherapy. Second-line or salvage treatment was administered to patients whose induction chemotherapy treatment was ineffective and who did not reach a complete response (CR) after consolidation therapy (**Figure 1**).

A total of 103 patients were enrolled from April 2015 to July 2018. The inclusion criteria were as follows: 1) patients with ND-ENKTL according to pathological examination; 2) survival prognosis of more than 3 months; and 3) ages 18–70 years. Exclusion criteria were as follows: 1) absence of risk factors in stage I; 2) severe cardiopulmonary, renal, or liver dysfunction; 3) inability to tolerate combined chemotherapy; and 4) pregnancy. All cases were assessed and diagnosed by 2 pathologists. The main diagnostic criteria were CD3+, CD56+, Epstein-Barr virus-encoded early RNA+, granzyme B+, and T-cell intracytoplasmic antigen-1+. Difficult or borderline cases were diagnosed after consultation with pathologists at another center. This study was reviewed, approved, and performed in compliance with the ethical standards of the ethics committee of Beijing Tongren Hospital (Approval No. TRECKY2015-016). Informed consent was obtained from each participant.

### Chemotherapy regimens

The SVILE regimen was implemented as follows: ifosfamide 1 g/m^2^ on days 1–3, IV; pegaspargase 2,500 U/m^2^ on day 2, IM; vindesine 4 mg on day 1, IV; etoposide 75 mg on days 1–3, IV; and dexamethasone 20 mg on days 1–4, IV. The P-GemOx regimen was implemented as follows: gemcitabine 1.2 g/m^2^ on day 1, IV; oxaliplatin 130 mg on day 1, IV; and pegaspargase 2,500 U/m^2^ on day 2, IV. The SVILE and P-GemOx regimens were repeated every 3 weeks.

In the event of AEs such as leukopenia or thrombocytopenia during the regimen, human recombinant granulocyte-colony stimulating factor (300 μg, once per day) and interleukin-11 (3 mg, once per day) were administered until the neutrophil and platelet counts returned to normal. If the fibrinogen decreased to < 1.0 g/L, human condensed fibrinogen or fresh frozen plasma was infused. Supportive treatments were administered appropriately when liver or kidney injury occurred. Antibiotics were administered in the event of fever and/or infection.

### Radiotherapy

Patients in stage I/II who achieved CR or partial response (PR) after 3 cycles of induction chemotherapy received further IFRT. Patients in stage III/IV who had limited lesions after induction chemotherapy received IFRT. The dose of IFRT ranged from 50 to 55 Gy. One month after radiotherapy, the same chemotherapy regimens were used to consolidate chemotherapy for 2 to 3 cycles. Generally, 2 or 3 cycles of consolidation chemotherapy were administered to patients who achieved CR or PR, respectively, after induction chemotherapy.

### Primary study endpoint

The primary study endpoint was the ORR (CR + PR) after 3 cycles of induction chemotherapy. The efficacy was evaluated with positron emission tomography/computed tomography (PET/CT, *n* = 66), enhanced CT (*n* = 21), and contrast magnetic resonance imaging (*n* = 16) after 3 cycles of induction chemotherapy. The evaluation methods showed no significant differences between the groups. Patients were divided into CR, PR, stable disease (SD), or progressive disease (PD) according to the Lugano standards^18^.

### Secondary study endpoints

The secondary study endpoints were CR rate after treatment, progression-free survival (PFS), and overall survival (OS) at the follow-up assessments. The patients were evaluated for treatment efficacy, and follow-up assessments were conducted every 3 months in the first year and every 6 months after the first year. Patient condition was evaluated in follow-up assessments, and the PFS and OS were recorded.

### Adverse events

During combined chemotherapy, all AEs were recorded and graded according to the Common Terminology Criteria for Adverse Events version 3.0 standards.

### Statistical analysis

The data were analyzed in SPSS version 16.0 software (IBM, Armonk, NY, USA). The ORR and CR rates were compared with a cross-tabulation χ^2^ test. The PFS and OS were estimated with Kaplan-Meier analysis. The statistical significance of differences between survival curves was evaluated with the log-rank test. The threshold for statistical significance was set at *P* < 0.05 with two-tailed tests.

## Results

### Patient characteristics

A total of 103 patients were enrolled, comprising 69 patients in stage I/II and 34 patients in stage III/IV. Patients were randomly divided into the experimental group (SVILE regimen, 52 cases) and the control group (P-GemOx regimen, 51 cases). There were no significant differences (*P* > 0.05) in the main characteristics between the groups (**Table 1**).

### Short-term responses

After 3 cycles of induction chemotherapy, the ORR values of the patients in the experimental group and control group were 86.5% and 88.2%, respectively. There was no significant difference (*P* > 0.05) in ORR between patients in stage I/II and stage III/IV. After the scheduled treatment protocol was completed, the CR rate did not significantly differ (*P* > 0.05) between the groups. There was no significant difference (*P* > 0.05) between the groups for stage I/II or for stage III/IV patients (**Table 2**).

### PFS and OS

**Figure 2:**
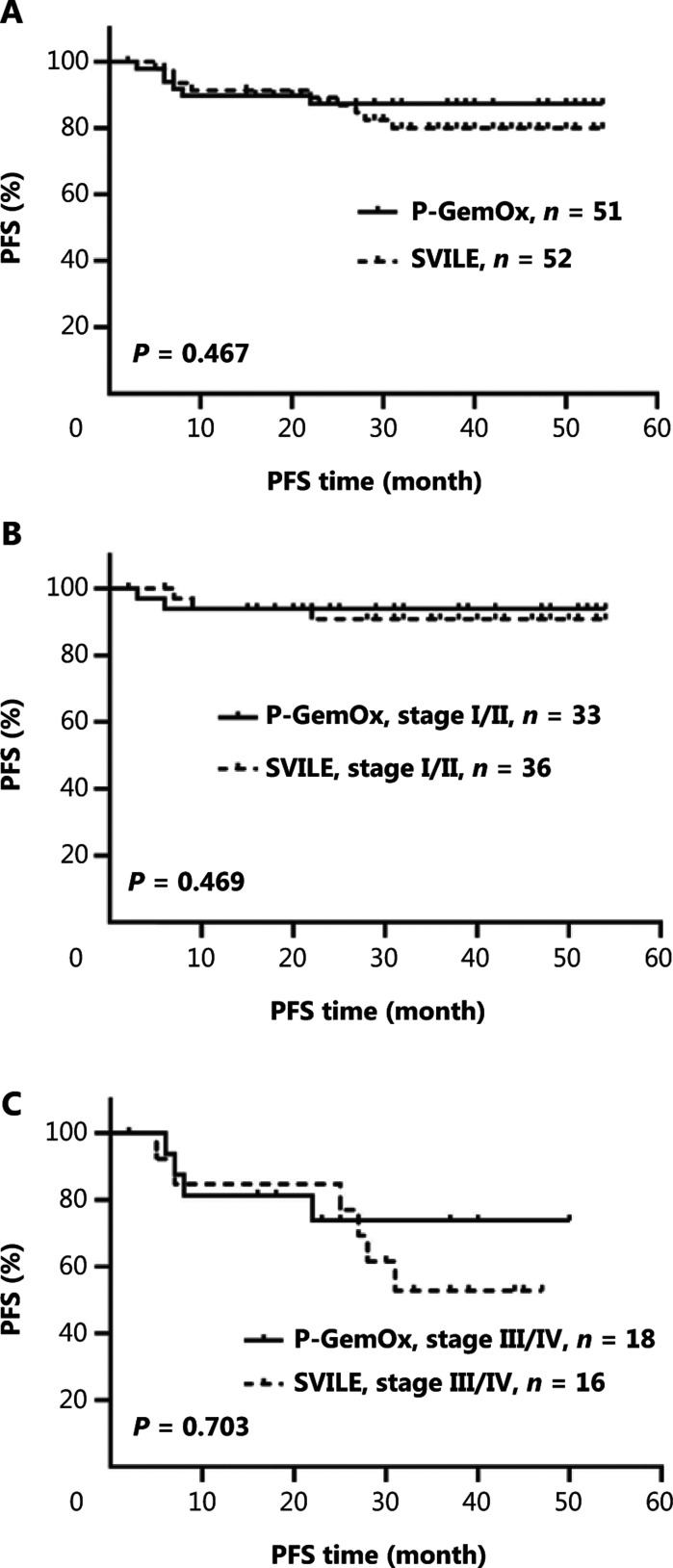
PFS of patients treated with the SVILE *vs.* P-GemOx regimen. (A) There was no significant difference in PFS between the experimental group and control group. The PFS of the experimental and control groups was, respectively, 86.1% *vs.* 86.3% at 1 year, 84.0% *vs.* 83.9% at 2 years, and 75.4% *vs.* 83.9% at 3 years. (B) There was no significant difference in PFS between patients in the experimental group and the control group in stage I/II. The PFS was 91.3% *vs.* 93.3% at 1 year, and 88.3% *vs.* 93.9% at both 2 and 3 years, respectively. (C) In stages III/IV, there was no significant difference in PFS between patients in the experimental group and control group, respectively, and the 1-year, 2-year, and 3-year PFS were 74.0% *vs.* 72.2%, 74.0% *vs.* 65.7%, and 46.2% *vs.* 65.7%. SVILE, ifosfamide, dexamethasone, pegaspargase, vindesine, and etoposide; P-GemOx, pegaspargase, gemcitabine, and oxaliplatin; PFS, progression-free survival..

**Figure 3:**
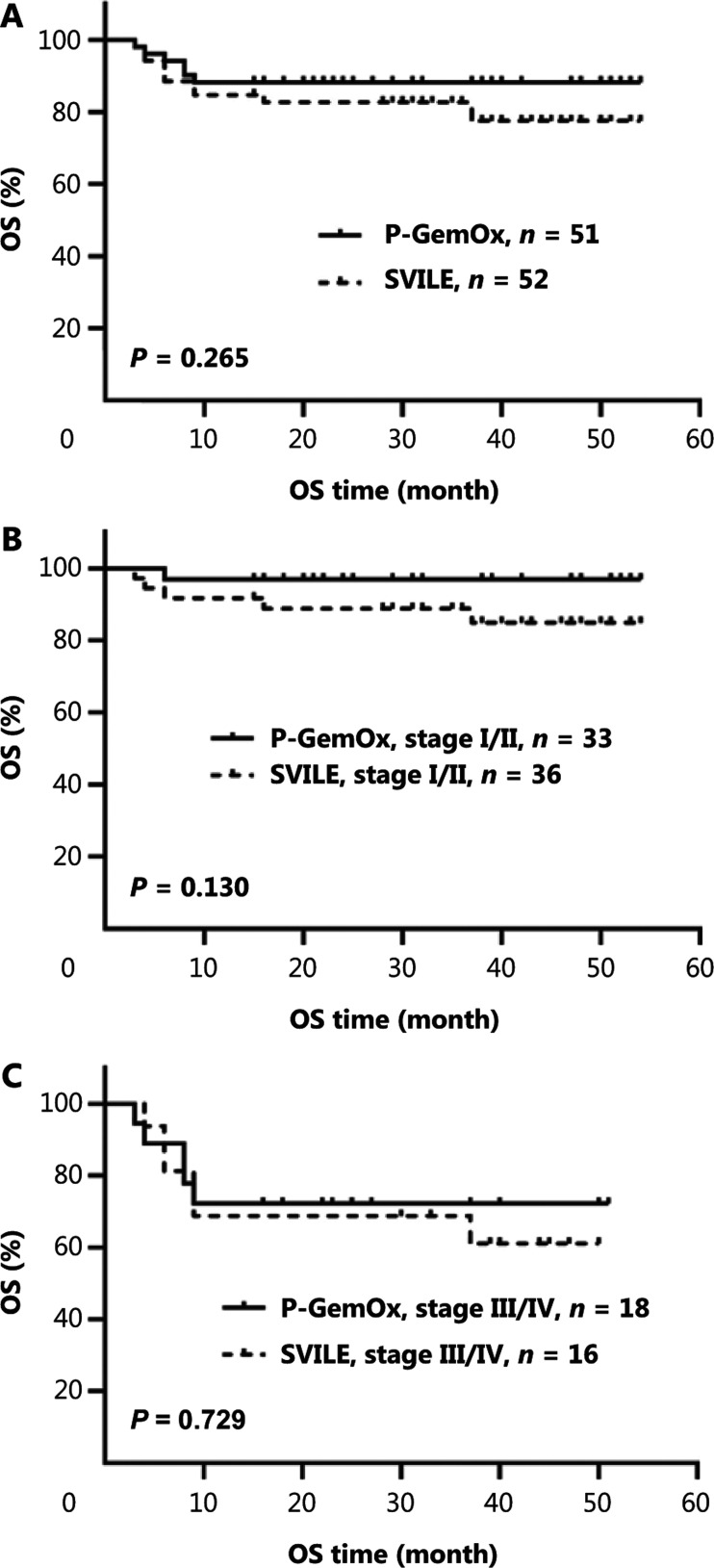
OS of patients treated with the SVILE or P-GemOx regimen. (A) There was no significant difference in OS between patients in the experimental group and control group. The OS in the experimental group and control group was, respectively, 84.6% *vs.* 88.2% at 1 year, 82.6% *vs.* 88.2% at 2 years, and 82.6% *vs.* 88.2% at 3 years. (B) There was no significant difference in OS between patients in the experimental group and the control group in stage I/II. The OS for the experimental group and control group was, respectively, 91.7% *vs.* 97.0% at 1 year, and 88.8% *vs.* 97.0% at both 2 and 3 years. (C) There was no significant difference in OS between patients in the experimental group and control group for stage III/IV, and the 1-year, 2-year, and 3-year OS were all 68.8% *vs.* 72.2%. SVILE, ifosfamide, dexamethasone, pegaspargase, vindesine, and etoposide; P-GemOx, pegaspargase, gemcitabine, and oxaliplatin; OS, overall survival.

Regular follow-up was conducted. The median follow-up times for the experimental and control groups were 30 and 29 months, respectively. There were no significant differences between the groups (*P* > 0.05) in overall PFS and OS. There were also no significant differences between PFS and OS (**Figures 2 and 3**).

### Adverse events

The incidence of common AEs in the experimental and control groups is listed in **Table 3**. There were no treatment-related deaths (grade 5 AE).

## Discussion

Chemotherapy regimens for ENKTL have recently improved. Anthracycline-containing treatments, such as CHOP and CHOP-like regimens, are no longer recommended for ENKTL, owing to the resultant high rate of multidrug resistance genes and Gp-170 proteins, which are resistant to anthracyclines^19^. Clinical trials have demonstrated that this regimen is not the first choice for patients with ENKTL^20^. L-asparaginase or pegaspargase-containing regimens have now become mainstream ENKTL treatments^10^.

Although the SMILE regimen has achieved good results in patients with advanced and refractory or relapsed ENKTL^21,22^, treatment-related toxicity is a serious concern. Therefore, SMILE is not recommended for patients in early stages of ENKTL. More recently, mSMILE has been studied for ENKTL treatment^23^; however, the high-dose methotrexate used in the SMILE and mSMILE regimens can penetrate the blood-brain barrier. Therefore, although these regimens provide better prevention and treatment of central nervous system lymphoma than alternative treatments, they are more toxic in patients with advanced or relapsed refractory ENKTL and require the administration of calcium folic acid, thus resulting in poor patient treatment tolerance. Additionally, according to Kim et al.^24^, the incidence of central nervous system invasion by ENKTL is low (5.76%). Therefore, we replaced methotrexate in the mSMILE regimen with vindesine, a vinca alkaloid, which is widely used for treating lymphoma, acute lymphocytic leukemia, and certain solid tumors^25^. We named this new treatment the SVILE regimen. Our control regimen was based on the commonly used PemOx/GELOX^26^, which has a positive effect on ENKTL. PemOx/GELOX treatment consists of pegaspargase, gemcitabine, and oxaliplatin, and is denoted P-GemOx. Similar treatment regimens have also achieved good results in the treatment of ND-ENKTL^11,27^.

We used the sandwich chemoradiation therapy modality to compare the short-term effects of the 2 regimens after 3 cycles of induction chemotherapy. P-GemOx treatment was more effective in the control group than SVILE was in the experimental group in terms of CR, but no significant differences were observed between groups. Previous studies have reported a CR rate of patients with early ENKTL after GELOX sandwich chemoradiation of 74%, and a total efficacy rate of 96%, values comparable to the results of our study. Li et al.^26^, in a retrospective multicenter study, have reported that the CR rate after treatment with gemcitabine, oxaliplatin, L-asparaginase, and dexamethasone (GELOXD)/gemcitabine, oxaliplatin, pegaspargase, and dexamethasone (P-GEMOXD) for patients with ND-ENKTL is 83%. In another retrospective study, the ORR and CR rates of patients with ND-ENKTL in stage I/II after receiving P-GEMOX sandwich chemoradiation have been found to be 94.3% and 80.0%, respectively. In several other studies, the CR rates in patients with stage I/II ENKTL have been reported to be between 81% and 89%^22,28–32^. In our prospective study, the ORR and CR rates of patients in the SVILE group and the P-GemOx group in stage I/II were fairly consistent with the results in the literature.

Previous studies have reported that the ORR of the SMILE regimen for stage IV and relapsed or refractory ENKTL can reach 79%, and the CR rate can reach 45%^21^. In another study, SMILE was used to treat 43 newly diagnosed and 44 refractory or relapsed patients with ENKTL. After 2 to 3 cycles of chemotherapy, the ORR was 78% (CR 56%, PR 22%). After a median of 3 cycles of chemotherapy (range 1–6), the ORR reached 81% (CR 66%, PR 15%)^22^. Likewise, another trial has reported a CR rate of 80% for the mSMILE regimens in ENKTL^33^. Although these treatment modalities differed slightly, the ORR was consistently between 70% and 80%. In other studies, treatment protocols have differed, and patients with advanced stage and relapsed or refractory ENKTL have been included; nonetheless, the treatment results are similar to those in our study^21,34–36^.

During follow-up assessments, we observed no significant differences in PFS and OS between the experimental and control groups in overall status, or in stage I/II or stage III/IV patients. Most patients with disease progression and death had SD or PD after induction therapy. Other studies have reported a 3-year PFS of patients with stage I/II ENKTL of 86% and a 3-year PFS of 72.8%^6,26^. In one study, the 2-year PFS and OS after SMILE sandwich chemoradiation therapy have been reported to be 83% and 100%, respectively^22^. Some reports have indicated that patients treated with L-asparaginase, cisplatin, etoposide, and dexamethasone chemoradiation have 3-year PFS and OS of 67% and 70%, respectively^30^. Additionally, patients treated with intensity-modulated radiation therapy followed by a GDP regimen have 3-year PFS and OS of 77% and 85%, respectively^31^. Most of these studies have been retrospective and have differed in treatment regimens; nonetheless, the results are generally similar to those of our study.

In our study, the 1-year follow-up PFS values in the experimental and control groups were similar to those reported in the literature for other treatment regimens. In one study, after 2 cycles of SMILE treatment, the 1-year PFS and OS were 53% and 55%, respectively^21^. In another case, after methotrexate, etoposide, dexamethasone, and pegaspargase chemotherapy, the 1-year PFS and OS were 62% and 69%, respectively^34^. P-GEMOX sandwich chemoradiation therapy has been reported to result in a 39% PFS and 65% OS at 2-year follow-up^35^. After GDP treatment, 1-year follow-up assessments have yielded PFS and OS of 55% and 73%, respectively^36^. In these studies, the treatment options and patients included were not consistent, and the results were somewhat different. In most cases, the prognosis of patients with stage III/IV is poor, with a 1-year PFS and OS of approximately 60%.

During chemotherapy, in this study, the most common AEs were hematological toxicity, hepatotoxicity, and coagulation abnormalities, which are known to result from the 2 chemotherapy regimens used. The slight differences in AEs were likely to be dependent on the regimen and, after timely supportive therapy was administered, they were swiftly resolved. All adverse effects were reversible and controllable. No grade 5 AEs (treatment-related deaths) occurred in this study.

SVILE is a newly designed chemotherapy regimen. In this prospective study, SVILE demonstrated similarly favorable therapeutic effects to those of the commonly used P-GemOx regimen for the treatment of patients with ND-ENKTL. The number of patients in the 2 groups was relatively small, and consequently further large-sample studies are needed to confirm the effects of the SVILE regimen in patients with ENKTL.

These 2 groups of patients are currently closely monitored. In the event of recurrence of the disease, lymphoma specimens will be tested for molecular biology-related indicators, and new treatments such as chemotherapy combined with monoclonal antibodies to programmed cell death protein 1 or programmed cell death ligand 1; histone deacetylase inhibitors; monoclonal antibody to CD38; or immunomodulator lenalidomide will be administered according to the molecular tests and literature findings^17^. To date, we have 2 clinical trials registered for relapsed and refractory ENKTL.

## Conclusions

In conclusion, the SVILE and P-GemOx regimens, combined with radiotherapy and consolidation chemotherapy (sandwich chemoradiation therapy), demonstrated positive short-term and long-term effects in the treatment of patients with ND-ENKTL. In particular, the novel SVILE regimen had positive effects, and the outcomes did not significantly differ from those of the commonly used P-GemOx regimen. Therefore, this SVILE regimen is worthy of further study.

## Figures and Tables

**Table 1:** Patient characteristics at enrollment

Characteristics	No. of patients (%)		*P*
	SVILE (*n* = 52)	P-GemOx (*n* = 51)	
Male	34 (65.4)	34 (66.7)	0.891
Age, median (range, years)	46 (18–63)	47 (18–67)	0.368
B-symptoms present	41 (78.8)	33 (64.7)	0.111
^†^PET/CT at diagnosis	51 (98.1)^a^	51 (100)	1.000
^‡^SUVmax, mean ± SD	11.727 ± 5.945	13.747 ± 7.232	0.117
Lymph node involvement	18 (34.6)	15 (29.4)	0.571
Nasal involvement	52 (100)	50 (98.0)^a^	0.495
Decreased serum album	19 (36.5)	13 (25.5)	0.226
Elevated serum ^§^LDH	12 (23.1)	13 (25.5)	0.775
Elevated serum ^¶^β-2 MG	28 (53.8)	24 (47.1)	0.491
Ann Arbor stage			
I/II	36 (69.2)	33 (64.7)	0.625
III/IV	16 (30.8)	18 (35.3)	
PINK grade			
Low	25 (48.1)	24 (47.1)	0.258
Intermediate	18 (34.6)	12 (23.5)	
High	9 (17.3)	15 (29.4)	
¥*-*IFRT	45 (86.5)	46 (90.2)	0.563

^†^PET/CT, positron emission tomography/computed tomography; ^‡^SUV, standard uptake value; ^§^LDH, lactate dehydrogenase; ^¶^β2 MG, beta-2 microglobulin; ¥-IFRT, involved-field radiotherapy; ^a^Fisher test was used when *n* < 5.

**Table 2:** Short-term responses in the experimental and control groups

Responses	No. of patients (%)		*P*
	SVILE	P-GemOx	
Responses by the end of induction ^†^CT	*n* = 52	*n* = 51	
^‡^CR	15 (28.8)	15 (29.4)	0.967
^§^PR	30 (57.7)	30 (58.8)	
^¶^SD/PD	7 (13.5)	6 (11.8)	
Responses of patients with stage I/II	*n* = 36	*n* = 33	
CR	14 (38.9)	13 (39.4)	0.789
PR	19 (52.8)	19 (57.6)	
SD/PD	3 (8.3)^a^	1 (3.0)^a^	
Responses of patients with stage III/IV	*n* = 16	*n* = 18	
CR	1 (6.2)^a^	2 (11.1)^a^	1.000
PR	11 (68.8)	11 (61.1)	
SD/PD	4 (25.0)^a^	5 (27.8)	
Responses by the end of treatment	*n* = 52	*n* = 51	
CR	41 (78.8)	43 (84.3)	0.802
PR	4 (7.7)^a^	2 (3.9)^a^	
SD/PD	7 (13.5)	6 (11.8)	
Responses of patients with stage I/II	*n* = 36	*n* = 33	
CR	30 (83.4)	32 (97.0)	0.236
PR	3 (8.3)^a^	0 (0)^a^	
SD/PD	3 (8.3)^a^	1 (3.0)^a^	
Responses of patients with stage III/IV	*n* = 16	*n* = 18	
CR	11 (68.8)	11 (61.1)	1.000
PR	1 (6.2)^a^	2 (11.1)^a^	
SD/PD	4 (25.0)^a^	5 (27.8)	

^†^CT, chemotherapy; ^‡^CR, complete response; ^§^PR, partial response; SD, stable disease; PD, progressive disease. ^a^Fisher test was used when *n* < 5.

**Table 3:** Adverse events in the SVILE and P-GemOx groups

Adverse event	Adverse event incidence, *n* (%)										*P*
	SVILE					P-GemOx					
Grade	0	1	2	3	4	0	1	2	3	4	
Hemoglobin	5 (9.6)	11 (21.1)	26 (50.0)	8 (15.4)	2 (3.8)^a^	8 (15.7)	13 (25.5)	25 (49.0)	3 (5.9)^a^	2 (3.9)^a^	0.533
Leukocytes	4 (7.7)^a^	5 (9.6)	16 (30.8)	17 (32.7)	10 (19.2)	4 (7.8)^a^	11 (21.6)	23 (45.1)	11 (21.6)	2 (3.9)^a^	0.037*
Neutrophils	5 (9.6)	3 (5.8)^a^	0 (0)	11 (21.2)	33 (63.5)	3 (5.9)^a^	5 (9.8)	10 (19.6)	19 (37.3)	14 (27.5)	0.000**
Platelets	36 (69.2)	8 (15.4)	7 (13.5)	0 (0)	1 (1.9)^a^	13 (25.5)	11 (21.6)	15 (29.4)	10 (19.6)	2 (3.9)^a^	0.000**
Fibrinogen	3 (5.8)^a^	11 (21.2)	23 (44.2)	15 (28.8)	0 (0)	3 (5.9)^a^	11 (21.6)	23 (45.1)	14 (27.5)	0 (0)	1.000
^†^PTT	11 (21.2)	19 (36.5)	15 (28.8)	7 (13.5)	–	7 (13.7)	24 (47.1)	19 (37.3)	1 (2.0)^a^	–	0.095
^‡^INR	50 (96.2)	2 (3.8)^a^	0 (0)	0 (0)	–	50 (98.0)	1 (2.0)^a^	0 (0)	0 (0)	–	1.000
Anorexia	11 (21.2)	27 (51.9)	14 (26.9)	0 (0)	0 (0)	12 (23.5)	24 (47.1)	15 (29.4)	0 (0)	0 (0)	0.885
Nausea	30 (57.7)	18 (34.6)	4 (7.7)^a^	0 (0)	0 (0)	31 (60.8)	15 (29.4)	5 (9.8)	0 (0)	0 (0)	0.830
Vomiting	43 (82.7)	4 (7.7)^a^	5 (9.6)	0 (0)	0 (0)	37 (72.5)	11 (21.6)	3 (5.9)^a^	0 (0)	0 (0)	0.131
^§^ALT	20 (38.5)	26 (50.0)	5 (9.6)	1 (1.9)^a^	0 (0)	8 (15.7)	24 (47.1)	13 (25.5)	6 (11.7)	0 (0)	0.006**
^¶^AST	20 (38.5)	24 (46.1)	7 (13.5)	1 (1.9)^a^	0 (0)	10 (19.6)	27 (52.9)	9 (17.6)	5 (9.8)	0 (0)	0.101
Bilirubin	43 (82.7)	5 (9.6)	4 (7.7)^a^	0 (0)	0 (0)	37 (72.5)	11 (21.6)	3 (5.9)^a^	0 (0)	0 (0)	0.261
Creatinine	50 (96.2)	2 (3.8)^a^	0 (0)	0 (0)	0 (0)	48 (94.1)	3 (5.9)^a^	0 (0)	0 (0)	0 (0)	0.678
Amylase	49 (94.2)	1 (1.9)^a^	2 (3.8)^a^	0 (0)	0 (0)	51 (100.0)	0 (0)	0 (0)	0 (0)	0 (0)	0.368

^†^PTT, partial thromboplastin time; ^‡^INR, international normalized ratio of prothrombin time; ^§^ALT, serum glutamic pyruvic transaminase, ^¶^AST: serum glutamic oxaloacetic transaminase. ^a^Fisher test was used when *n* < 5. *P < 0.05; **P < 0.01.
